# Disappointment and frustration, but long-term satisfaction: patient experiences undergoing treatment for a chronic Achilles tendon rupture—a qualitative study

**DOI:** 10.1186/s13018-022-03103-7

**Published:** 2022-04-09

**Authors:** Anna Nordenholm, Niklas Nilsson, Ferid Krupic, Eric Hamrin Senorski, Katarina Nilsson Helander, Olof Westin, Jón Karlsson

**Affiliations:** 1 Department of Health and Rehabilitation, Institute of Neuroscience and Physiology, Sahlgrenska Academy, University of Gothenburg, Universitetsplatsen 1, 405 30 Gothenburg, Sweden; 2 Department of Orthopedics, Institute of Clinical Sciences, Sahlgrenska Academy, University of Gothenburg, Gothenburg, Sweden

**Keywords:** Delayed treatment, Missed diagnosis, Late detected, Patient perspective, Interview

## Abstract

**Background:**

Delayed treatment of Achilles tendon ruptures is generally due to either misdiagnosis or patient delay. When the treatment is delayed more than 4 weeks, the rupture is defined as “chronic”, and almost always requires more invasive surgery and longer rehabilitation time compared with acute Achilles tendon ruptures. There is insufficient knowledge of patient experiences of sustaining and recovering from a chronic Achilles tendon rupture.

**Methods:**

To evaluate patients’ experiences of suffering a chronic Achilles tendon rupture, semi-structured group interviews were conducted 4–6 years after surgical treatment using a semi-structured interview guide. The data were analyzed using qualitative content analysis described by Graneheim and Lundman.

**Results:**

The experiences of ten patients (65 ± 14 years, 7 males and 3 females) were summarized into four main categories: (1) “The injury”, where the patients described immediate functional impairments, following either traumatic or non-traumatic injury mechanisms that were misinterpreted by themselves or the health-care system; (2) “The diagnosis”, where the patients expressed relief in receiving the diagnosis, but also disappointment and/or frustration related to the prior misdiagnosis and delay; (3) “The treatment”, where the patients expressed high expectations, consistent satisfaction with the surgical treatment, and addressed the importance of the physical therapist having the right expertise; and (4) “The outcomes”, where the patients expressed an overall satisfaction with the long-term outcome and no obvious limitations in physical activity, although some fear of re-injury emerged.

**Conclusions:**

An Achilles tendon rupture can occur during both major and minor trauma and be misinterpreted by both the assessing health-care professional as well as the patient themselves. Surgical treatment and postoperative rehabilitation for chronic Achilles tendon rupture results in overall patient satisfaction in terms of the long-term outcomes. We emphasize the need for increased awareness of the occurrence of Achilles tendon rupture in patients with an atypical patient history.

**Supplementary Information:**

The online version contains supplementary material available at 10.1186/s13018-022-03103-7.

## Background

The overall incidence of Achilles tendon rupture (ATR) is reported to be 12–47 per 100,000 person-years and most commonly occurs in men around 40 years of age [1–4]. The majority of ATRs occur in conjunction with high-intensity sport activities and are characterized by a loud “pop”, followed by intensive ankle pain [5, 6]. However, up to 25% of ATRs are reported to occur during minor trauma, not related to sporting activities [7]. The symptoms associated with ATR include pain, an immediately affected gait pattern, recurrent swelling, and an inability to perform adequate heel rises [8, 9]. Diagnosis is based on patient history and physical examination including clinical tests with high sensitivity and specificity for detecting an acute ATR, such as Thompson’s test (Calf squeeze test) and Matle’s test [10, 11].

Patients presenting with an atypical patient history are less likely to seek early medical attention and are, when seeking medical attention, often not clinically assessed as thoroughly as patients describing a classical trauma followed by intense ankle pain [12, 13]. Due to patient delay or inadequate clinical examination by the assessing health-care professional, up to 10–25% of all ATRs are reported to be missed in the acute phase of the injury [7, 14]. The missed diagnosis results in delayed treatment and a risk of the rupture becoming chronic. Chronic ruptures require more invasive surgery and may result in longer rehabilitation times and longer absence from work and sporting activity than if the ATR had been treated acutely [7]. Chronic Achilles tendon ruptures (CATR) are defined as an ATR with delayed treatment more than 4 weeks after the initial injury and are most common in individuals older than 60 years [13, 15, 16].

Surgical intervention improves patient-reported function and objective foot- and ankle function in patients with CATR, however, there is a large variation in outcomes between patients [7, 8, 17, 18]. The existing literature suggests that impairments in ankle-related function are common and persist several years after the surgical treatment of CATR [7, 17]. In addition to quantitative measures of subjective and objective outcome, studies with a qualitative approach enable a deeper understanding of the patient’s own experiences and highlight possible areas of improvement in surgical treatment and rehabilitation. To our knowledge, no previous study has qualitatively investigated the experience of sustaining a CATR from the patient’s perspective, which is a feature of great importance to incorporate in outcome research. Therefore, semi-structured group interviews investigating experiences related to the injury event, the diagnosis and long-term outcomes were conducted.

## Methods

### Study design and participants

Qualitative group interviews were conducted using a semi-structured interview guide consisting of open-ended questions regarding the patient’s experiences of sustaining a CATR (Additional file 1: Appendix 1). The g included questions on the injury, interactions with the health-care system, current foot- and subjective ankle-related function, and thoughts about the future. The data were analyzed using qualitative content analysis described by Graneheim and Lundman [19] with interpretation of the manifest data.

In addition, data on the patient’s age and occupation were collected. The Consolidated criteria for Reporting Qualitative research (COREQ) [20] was used to report methodological information.

Patients with CATR that had previously participated in another, still unpublished study conducted by our research group, were contacted by phone, by either AN or NN, and invited to participate (*n* = 24). The previous study had included Swedish speaking patients with CATR, defined as an Achilles tendon rupture left untreated for more than 4 weeks. All patients were scheduled for surgical intervention at Sahlgrenska University Hospital (Gothenburg, Sweden) or Kungsbacka Hospital (Kungsbacka, Sweden) between 2014 and 2016. All patients were treated with augmentation with a free flap from the gastrocnemius aponeurosis, a surgical technique previously presented by Nilsson-Helander et al. [21]. Twenty-two of the twenty-four eligible patients were reached and asked if they wanted to participate in an interview study examining patient experiences of CATR, of which eleven agreed to participate (eight males and three females; mean age 65 ± 14 years, median 63 years). Of the 13 patients, who were not included, mean age was 53 (range 34–80) and 38% were females. Two of the patients were not reached, four declined to participate, and seven were interested but were unavailable to participate due to practical reasons.

Prior to agreement to participate, the patients received oral and written information of the purpose and method of the study and the occupation of those conducting the study. The patients were informed about potential risks and benefits of the study and that participation was voluntarily and they could withdraw from the study at any time without any explanation. Informed consent was obtained from each participant prior to the start of the study. Ethical approval was obtained by the Regional Ethical Review Board in Gothenburg (reference number 554-15, 2015-09-30, supplementary approval 2019-06-10).

### Data collection

The interview guide was drafted by the first (AH) and second authors (NN), both with experience working with orthopedic patients (Additional file 1: Appendix 1). The guide was then confirmed by the third author (FK). Four group interviews with open-ended questions were performed between October 2019 and December 2020 in groups of 2–4 patients. At the time of data collection, between 4 and 6 years had passed since surgical treatment. Authors AH and NN were present during all four interviews, and the third author FK during the first interview session. The first three interviews were held face-to-face by AH and NN in a conference room at the research facility. The fourth interview was held by AH and NN through video conference due to the COVID-19 pandemic (Zoom Video Communications). All interviews began with the interviewers introducing themselves and repeating background information and the purpose of the study. The interviewer then asked open-ended questions according to the interview guide and followed up with questions such as “How did you experience that? What were your thoughts/feelings?”. After four group interviews that lasted between 21 to 55 min had been conducted, saturation was considered to be achieved. While conducting the interviews, one patient was found to have had received treatment less than 4 weeks after the injury and was therefore incorrectly included in the study. The patient was excluded from the analysis, resulting in a total of ten patients completing the study. All interviews were audio recorded in Swedish, with the patients participating anonymously. The recordings were later transcribed verbatim by an external person.

### Data analysis

The data were analyzed using qualitative content analysis described by Graneheim and Lundman with interpretation of manifest data [19]. Qualitative content analysis focuses on the subject and the context, and is characterized by finding similarities and differences within codes and categories. In each step of the analysis, AN and NN first performed separate analyses, which were then compiled through systematical discussions. In the first step, the transcribed text was read thoroughly to obtain understanding of the data and then shortened through condensation. Subsequently, the condensed text was abstracted using codes, which in turn were sorted into sub-categories and main categories through a process of reflection and discussion between the authors. The results of the analysis were read and confirmed by FK. The sub-categories, constituting the manifest content, were then formulated into main categories covering the content of the sub-categories. The citations in the text were translated by AN and the final manuscript was drafted by AN, NN, FK, EHS, KNH, OW and JK. All authors read and approved the final manuscript.

## Results

The ten patients’ experiences of sustaining a CATR were summarized into four main categories, i.e. (1) The injury, (2) The diagnosis, (3) The treatment, and (4) The outcome. The main categories and sub-categories are summarized in Table 1.

**Table 1:** Main categories and sub-categories

Main categories	The injury	The diagnosis	The treatment	The outcomes
Sub-categories	Varying injury mechanism	Feeling of relief	High expectations	Satisfaction with outcomes
	Persisting pain and disabilities	Disappointment and frustration	Satisfaction with treatment	No negative thoughts about the future
	Delay of treatment			

### The injury

The patients in the present study described varying injury mechanisms and reasons for the delayed treatment. They described traumatic as well as non-traumatic and non-specific injury-events, occurring during sporting activities as well as during low demanding every day activities. All of them experiences immediate functional disabilities and described it as the ankle was not working as normal, which affected their walking ability. Common to all was that they did not understand that the Achilles tendon had ruptured and some of them awaited to seek medical attention as they thought that the symptoms would be transient. Others sought medical attention but were misdiagnosed. The unsuccessful treatments had the patients confused and the longer the time went by they understood that something was not right.

#### Varying injury mechanisms

There was an occurrence of *varying injury mechanisms* between the patients, ranging from a clear event of injury to no known time point of the rupture (Table 2). Three out of ten patients could not define a specific time-point for the injury and one patient stated that the tendon most likely ruptured gradually. The patients that had experienced a traumatic injury mechanism described a sudden pain with subsequent swelling and in some of the cases a loud “snap” was described. The patients described immediate impairments when the rupture had occurred, which did not get better with time despite rest or rehabilitation.

“I had recently had surgery on both knees and had walked with those big orthoses on... and I just walked over the threshold at home in the kitchen and heard a bang and oh how weird I thought... I thought I sprained my ankle since I had had plaster cast for such a long time on both knees but it did not get better”

“I happened to… those lines (ropes) that split the boule courts, stick my foot into that and got a loop around my foot without noticing… then when I were to take a step, I put my other foot on the rope with resulted in that there was no step, instead I just fell forward and it, eh was really painful”

**Table 2:** Patient characteristics, injury mechanisms and reasons for delayed treatment

Patient	Age	Sex	Occupation	Injury mechanism	Reasons for delayed treatment
1	70	Male	Retired	Cannot with certainty account for a specific time-point of injury. Retrospectively suspects the injury happened in conjunction with using a mechanical cultivator	Waited to seek care a few months believing it was a tendinitis, which he had previously had on the contralateral side
2	78	Male	Retired	Sprained his ankle while playing with grandchildren on the beach	Waited to seek care a few weeks believing it was only an ankle sprain. Received the diagnosis partial rupture of the Achilles tendon at the first medical assessment in primary care and were referred to physical therapy
3	75	Female	Retired	Stumbled over a threshold at home	Waited to seek care believing it was an ankle sprain
4	72	Male	Retired	Cannot with certainty account for a specific time-point of injury	Waited to seek care a few months believing it was a tendinitis
5	75	Female	Retired	Cannot with certainty account for a specific time-point of injury. Retrospectively suspects the rupture occurred gradually, first partially when climbing up from the water on slippery cliffs and finally a few months later when walking down the stairs	Sought medical attention in the primary care 1 week after the final injury event and received the diagnosis ankle sprain
6	69	Female	Retired	Stumbled on an obstacle on the ground and fell forward	Waited to seek care a few months because of busy at work. Sought medical attention in primary care a few weeks after and received the diagnosis calf muscle tear
7	74	Male	Working	Forced dorsiflexion of the ankle during obstacle course training	Received only the diagnosis ligament tear at the acute setting
8	77	Male	Working	Playing badminton for the first time in 40 years	Achilles tendon rupture was ruled out by medical assessment in primary care and the patient were referred to physical therapy
9	52	Male	Working	Threw himself backwards during a gas explosion	Waited to seek care not realizing the extent of the injury
10	33	Male	Working	Forced dorsiflexion of the ankle during obstacle course training	Waited to seek care for a week believing it was a calf muscle tear. Received the diagnosis calf muscle tear when seeking medical attention in primary care

#### Persisting disabilities

The most commonly described *persisting disabilities* after injury and prior to treatment included pain, imbalance, and limping. As two of the patients reported:

“I went to exercise and tried to get going again … ehm…but I limped and, in the end (prior to the correct diagnosis and treatment), basically just dragged my leg behind myself… and thought what the hell, how weak am I really ... It didn’t get better …”

“I saw a physical therapist and trained etcetera, but the foot lagged behind and ... all the bodyweight was put on the right (non-injured) side, the left side didn’t work… I tried to load it ... and stand on my tip-toes but I couldn’t make it, I just fell down …”

#### Delayed treatment

The reason for *delayed treatment* of the injury varied between the patients; either due to the patients themselves had waited to seek health-care or that the injury was incorrectly diagnosed by the assessing physician. Suspected recurring Achilles tendinopathy or just a minor injury emerged as reasons in cases where the patients themselves were responsible for the treatment delay. They stated that since they did not understand the extent of the injury, they tried to self-medicate with non-steroidal anti-inflammatory drugs (NSAIDS) and/or waited to seek medical attention.

Ankle sprain, a partial tear of the tendon or calf muscle tear, were incorrect diagnoses the patients had received by the assessing health-care professionals. In one patient who had sustained a trauma during a sports activity, an ankle ligament injury was found during assessment, but the ATR was missed. One patient had received both a cortisone injection and shockwave therapy without results. A recurring topic was that the patients felt as if it was up to themselves to ensure that they received the right help. A feeling of having to convince health-care representatives that the injury required further assessment also emerged.

“… but well, it is simply that we live in a time at the moment where you have to be healthy to be sick… ehm … you have to be healthy, eloquent, speak up for yourself and be assertive… because otherwise you get nothing”

### The diagnosis

The lack of improvement over time got the patients frustrated and resulted in new contact with health care. To finally receive the correct diagnosis, and thereby opportunity to treatment, was a relief. However, the patients also expressed disappointment in relation to the competence of the health care professionals.

#### Feeling of relief

The patients agreed that it was *a relief* to receive a correct diagnosis, so that they could get an answer to why there had been an absence of improvement since the injury and to receive an appropriate treatment.

“It was nice (to get the right diagnosis) … haha … because I had dragged my leg for a long time and thought it was a calf muscle rupture and started to think about why it did not get better … so it was quite nice just to get an answer to what was wrong … ehm … so I guess it felt good”

“I was not particularly surprised because it became quite obvious the longer the time actually went that something was very wrong …”

#### Disappointment and frustration

The patients expressed “no hard feelings” against health-care representatives for getting an incorrect diagnosis, as well as a feeling of *disappointment and frustration* regarding that the assessing health-care professional did not have the capacity to see that the tendon was ruptured. One patient expressed frustration over that 1 year of doing rehabilitation had been for nothing. The opinion about lack of competence in primary care, the setting where they had received the incorrect diagnosis, was recurring among the patients. One patient who had received an incorrect diagnosis expressed that:

“I was pissed and bitter for quite some time … and went all into the mode that (i.e. bitterness regarding), ok, too much money should not be spent on (medical) examinations … those extra tests … because it’s expensive and not financially defensible … that was how I felt for quite some time …

### The treatment

Common to all was having high expectations on the outcomes prior to surgical treatment and they had confidence in the treating health care professionals. Factors that emerged as being important from the patient perspectives were to, as a patient, be devoted to rehabilitation and that the health care professionals had diagnosis-specific skills to treat Achilles tendon ruptures.

#### High expectations

The patients expressed that they had *high expectations* of the outcomes prior to surgery. They had high confidence in the health-care specialists and expected a good recovery referring to friends whom had undergone surgery for Achilles tendon rupture with good results.

“I expected to be okay again, to be able to getting started again”

“It was not up for discussion; I would recover well… I am an optimist, a previous sports girl… there was only one level, good”

#### Satisfaction with treatment

The patients were very *satisfied* with the orthopedic surgeon and her/his team, but there were some variations in degree of satisfaction with the physical therapist. The physical therapist having specific expertise in rehabilitation of ATR emerged as something the patients considered being important. The patients who had seen a physical therapist with previous experience of ATR expressed satisfaction with that fact and also appreciation of there being a dialogue between the orthopedic surgeon and physical therapist when needed. Moreover, when asked of areas that could be improved in their treatment, the patients requested more specific recommendations and information of where to find specialized physical therapists in primary care.

“I got the same rehabilitation program I previously had for my knee… I don’t think it was specific for improving the Achilles tendon… instead it was a general program that I got from them”

“I was recommended to see a physiotherapist with special expertise in Achilles tendons that was absolutely fantastic… she was my rescue"

### The outcomes

The patients expressed an overall satisfaction with the long-term outcomes after treatment. They had no functional limitations that restricted them in physical activity and did not think the injury would affect them negatively in the future. However, a fear of re-injury appeared, which had them refraining from activities such as running.

#### Satisfaction with outcomes

The current level of physical activity varied between patients from daily life activities only, to exercising at the gym or running. Remaining injury-related symptoms that emerged were stiffness and sensitivity loss at the ankle and foot. The patients expressed an overall *satisfaction* with the final outcome of treatment. None of them expressed any obvious limitations in physical activity, however, it emerged an avoidance of running due to fear of re-rupture.

“Maybe I am a bit too careful… but I have chosen not to run”

#### No negative thoughts about the future

Although a lingering fear of re-injury was expressed, none of the patients believed that the injury would affect them negatively in the future.

“As I see it… if it happens (re-injury) it happens… as I feel today I don’t think it will”

## Discussion

The purpose of this study was to explore patient experiences of sustaining and recovering from a chronic Achilles tendon rupture. The patient experiences were summarized into four main categories: (1) The injury; (2) The diagnosis; (3) The treatment and (4) The outcomes. There were inconsistencies among the patients in terms of the injury event, the injury mechanism, and their own perception of what had happened. These variations are in line with the findings of a previous study by Fridén et al., who investigated all error events and complaints related to delayed diagnosis and treatment of ATR that were reported to The Health and Social Care Inspectorate in Sweden [13]. In the study by Fridén et al. some of the injuries were characterized as non-specific injury events [13]. They also reported that the Achilles tendon had ruptured gradually rather than momentarily in some cases, as concluded by the assessing health-care professional. In the present study, some patients could not define a specific time-point for the injury and experienced immediate or gradually increasing pain, swelling and trouble with push-off while walking.

A shared experience among the patients was that the functional deficits did not improve as expected, which eventually led to them seek help from the health-care system. Achilles tendinopathy, ankle distortion, and partial tendon or muscle tear were common misdiagnoses, both in the cases where the injury was misinterpreted by the patient and when the wrong diagnosis was given by a health-care professional. These differential diagnoses have previously been described as the most common misdiagnoses in patients with ATR since they can cause similar disabilities [22]. However, ATR is a clinical diagnosis which is based on a comprehensive patient history and high-quality clinical tests. Both Thompson’s test and Matle’s test have a high sensitivity and specificity for detecting an acute ATR, and should always be performed to exclude an ATR in patients seeking medical attention complaining of difficulty with push off, poor balance and/or pain located around the foot, ankle or lower leg [10, 11]. The study by Fridén et al. revealed that an incomplete clinical examination had been carried out in the majority of the error events [13].

A feeling of relief when receiving the ATR diagnosis was expressed by the patients. However, disappointment related to the delayed recovery also emerged. Frustration and, from their view, the opinion of a lack of competency in primary health-care for diagnosing acute injuries came up repeatedly. Once they had received the diagnosis, the patients agreed on having high expectations of the upcoming surgical treatment. There was a consistent satisfaction with the surgeon, physical therapist as well as the long-term outcomes of the treatment, despite some persistent functional limitations related to the CATR. However, we do not know if satisfied patients chose to participate in the study to a larger extent than did unsatisfied patients. The degree of trust in the treating health-care professional is an important factor in the caregiver–patient relationship and may have a profound impact on patient self-reported health outcome and satisfaction with treatment [23]. In line with the findings in the present study, previous literature shows an overall good patient-reported outcome both in the short and long term after surgical treatment for CATR [17]. None of the patients related their persistent functional limitations to reduced muscle function, although previous literature reports that persistent reductions in calf muscle strength and heel-rise height are common after CATR [17]. Rather, the patients related limitations in physical activity to a fear of re-injury.

In addition to greater patient suffering by an extended recovery time, delayed treatment of ATR also results in negative health-economic consequences, as shown in a previous study conducted by our research group [24]. The mean health-care costs for treatment of CATR are considerably higher compared with surgically and non-surgically treatment of ATR. When affecting working patients, CATR may also generate higher production loss cost compared to ATR. The majority of cases of misdiagnosis in this study, as well as in the study by Fridén et al. [13], occurred in the primary health-care setting, which strongly indicates the need for more frequent use of clinical tests for ruling out an ATR. The strong association between ATR and sports-related trauma is problematic, since this is not always true and can, demonstrably, mislead both health-care professionals and patients. Greater awareness must be achieved, both in the society and in health-care, with regards to the presence of atypical patient-histories and also the occurrence of ATR in older populations. Fridén et al. reported that delayed treatment of ATR commonly occurs in patients > 60 years of age and that more than one-fourth of the patients were using medications known for increasing risk of tendon pathology as a side effect [13].

The present study provides with new perspectives on CATR and the outcomes of surgical treatment. The findings in this study can be used to complement and refine quantitative studies on the subject. One limitation of data collection through group interviews may result in some of the participants dominating the conversation more than others, and that the participants’ answers may be influenced by others. All non-verbal communication was missed in the data analysis process since the interviews only were audio recorded and the transcripts were not returned to the participants for comments or corrections. Data collected on few individuals cannot be generalized to a larger population, but may be transferrable to another similar setting. The patients included in the present study received similar surgical treatment and were living in the same geographic area, thus, the generalizability of the findings to patients treated with other surgical methods or patients in other countries may be limited.

## Conclusions

Achilles tendon ruptures can occur during sporting activities as well as daily activities and can be misinterpreted both by health-care professionals and the patients themselves. Irrespective of reason for delayed treatment, receiving the correct diagnosis is a relief. The reveal of a previous misdiagnosis also leads to disappointment and frustration among patients. Expectations prior to surgery are high and there is an overall satisfaction with outcomes after surgical treatment and rehabilitation of CATR. The results emphasize a need of greater awareness, both in society and among health-care professionals, with regards to the presence of ATR presenting with an atypical patient history with minor trauma and also the occurrence of ATR in the older populations.

## Supplementary Information

**Additional file 1: Appendix 1**. Interview guide.

## Data Availability

The datasets used and/or analyzed in the current study are available from the corresponding author on reasonable request.

## Abbreviations

ATR: Achilles tendon rupture
CATR: Chronic Achilles tendon rupture
NSAID: Non-steroidal anti-inflammatory drug

## References

[1] Ganestam A, Kallemose T, Troelsen A, Barfod KW (2016). Increasing incidence of acute Achilles tendon rupture and a noticeable decline in surgical treatment from 1994 to 2013. A nationwide registry study of 33,160 patients. Knee Surg Sports Traumatol Arthrosc.

[2] Huttunen TT, Kannus P, Rolf C, Felländer-Tsai L, Mattila VM (2014). Acute Achilles tendon ruptures: incidence of injury and surgery in Sweden between 2001 and 2012. Am J Sports Med.

[3] Čretnik A, KošKir R, Kosanović M (2010). Incidence and outcome of operatively treated Achilles tendon rupture in the elderly. Foot Ankle Int.

[4] Järvinen TA, Kannus P, Maffulli N, Khan KM (2005). Achilles tendon disorders: etiology and epidemiology. Foot Ankle Clin.

[5] Jozsa L, Kvist M, Balint BJ, Reffy A, Jarvinen M, Lehto M (1989). The role of recreational sport activity in Achilles tendon rupture. A clinical, pathoanatomical, and sociological study of 292 cases. Am J Sports Med.

[6] Movin T, Ryberg A, McBride DJ, Maffulli N (2005). Acute rupture of the Achilles tendon. Foot Ankle Clin.

[7] Maffulli N, Via AG, Oliva F (2017). Chronic Achilles tendon rupture. Open J Orthop.

[8] Kraeutler MJ, Purcell JM, Hunt KJ (2017). Chronic Achilles tendon ruptures. Foot Ankle Int.

[9] Jones MP, Khan RJ, Carey Smith RL (2012). Surgical interventions for treating acute Achilles tendon rupture: key findings from a recent cochrane review. J Bone Jt Surg.

[10] Reiman M, Burgi C, Strube E, Prue K, Ray K, Elliott A (2014). The utility of clinical measures for the diagnosis of Achilles tendon injuries: a systematic review with meta-analysis. J Athl Train.

[11] Maffulli N (1998). The clinical diagnosis of subcutaneous tear of the Achilles tendon. Am J Sports Med.

[12] Maffulli N (1999). Rupture of the Achilles tendon. J Bone Jt Surg.

[13] Fridén T, Movin T, Andrén-Sandberg Å. Missed diagnosis of Achilles tendon ruptures most common in elderly patients. Lakartidningen. 2017;114. 28535025

[14] Carden DG, Noble J, Chalmers J, Lunn P, Ellis J (1987). Rupture of the calcaneal tendon. The early and late management. J Bone Surg.

[15] Maffulli N, Ajis A, Longo UG, Denaro V (2007). Chronic rupture of tendo Achillis. Foot Ankle Clin.

[16] Gabel S, Manoli A (1994). Neglected rupture of the Achilles tendon. Foot Ankle Int.

[17] Hadi M, Young J, Cooper L, Costa M, Maffulli N (2013). Surgical management of chronic ruptures of the Achilles tendon remains unclear: a systematic review of the management options. Br Med Bull.

[18] Maffulli N, Oliva F, Maffulli GD, Buono AD, Gougoulias N (2018). Surgical management of chronic Achilles tendon ruptures using less invasive techniques. Foot Ankle Surg.

[19] Graneheim UH, Lundman B (2004). Qualitative content analysis in nursing research: concepts, procedures and measures to achieve trustworthiness. Nurse Educ Today.

[20] Tong A, Sainsbury P, Craig J (2007). Consolidated criteria for reporting qualitative research (COREQ): a 32-item checklist for interviews and focus groups. Int J Qual Health Care.

[21] Nilsson-Helander K, Sward L, Silbernagel KG, Thomee R, Eriksson BI, Karlsson J (2008). A new surgical method to treat chronic ruptures and reruptures of the Achilles tendon. Knee Surg Sports Traumatol Arthrosc.

[22] Ballas M, Tytko J, Mannarino F (1998). Commonly missed orthopedic problems. Am Fam Physician.

[23] Birkhäuer J, Gaab J, Kossowsky J, Hasler S, Krummenacher P, Werner C (2017). Trust in the health care professional and health outcome: a meta-analysis. PLoS ONE.

[24] Nilsson N, Nilsson Helander K, Hamrin Senorski E, Holm A, Karlsson J, Svensson M (2020). The economic cost and patient-reported outcomes of chronic Achilles tendon ruptures. J Exp Orthop.

