# Supplementary material for: Disappointment and frustration, but long-term satisfaction: patient experiences undergoing treatment for a chronic Achilles tendon rupture—a qualitative study
Source: J Orthop Surg Res. 2022 Apr 9;17:217. doi: 10.1186/s13018-022-03103-7 (PMC8994186; doi:10.1186/s13018-022-03103-7)
Supplement: Supplementary file 1 — Additional file 1: Appendix 1. Interview guide. [file 13018_2022_3103_MOESM1_ESM.docx]

# Additional file 1: Appendix

# Interview guide

**Theme 1: The injury**

Can you tell us about the injury event?

**Theme 2: The contact with health-care**

Can you describe the process when you first sought medical attention?

What was your experience of receiving the correct diagnosis?

What were your expectation on undergoing surgical treatment?

Did you have any fears prior to treatment?

What is your experience of how the health-care handled your injury?

In your opinion, could the health-care have done something different?

Do you have any positive experiences of the health-care?

**Theme 3: Impact on life**

How does the Achilles tendon rupture affect your daily life today?

Do you think that the injury will affect you in the future? And if so, how?

**Follow-up questions**

How did you experience that?

How did that make you feel?

What were your thoughts at that point?
